# Extracorporeal shock wave therapy for treating primary dysmenorrhea: A randomized controlled trial

**DOI:** 10.1097/MD.0000000000023798

**Published:** 2021-02-05

**Authors:** Ruirui Xing, Jian Yang, Renwei Wang, Yan Wang

**Affiliations:** a School of Kinesiology, Shanghai University of Sport; b Department of Rehabilitation, Shanghai Xuhui Central Hospital, Shanghai, China.

**Keywords:** acupuncture point, menstrual pain, primary dysmenorrhea, prostaglandin, radial extracorporeal shock wave therapy

## Abstract

**Background:**

There are scanty data to apply radial extracorporeal shock wave therapy (rESWT) on the acupuncture points in the lower abdomen to reduce the menstrual pain. This trial aimed to test the rESWT safety and efficacy for treating primary dysmenorrhea (PD).

**Methods:**

Forty-four young-women with PD were randomly assigned to one of the three groups: to receive rESWT on the acupuncture points during the follicular phase (Group A, n = 15) or during the luteal phase (Group B, n = 14), or to apply heat patch to the acupuncture points during the follicular phase as the control (Group C, n = 15) over three menstrual cycles. The pain severity (using 0-to-10 visual analog scale), the pain duration (hours), plasma PGF_2α_ prostaglandin F2alpha and prostaglandin E2 (PGE_2_), self-rating anxiety scale and menstrual blood loss were assessed before and after interventions.

**Results:**

The pain severity and duration significantly decreased in all groups after interventions. Although the reduced pain duration was not different among the groups, the reduced pain severity was more significant (*P* = .003) in Groups A (−53.8 ± 33.7%) and B (−59.3 ± 36.7%) than in Group C (−18.7 ± 27.1%). The rESWT intervention did not change plasma prostaglandins in Group A, although there was a decreased prostaglandin F2alpha (−20.5 ± 32.9%) in Group B or a decreased PGE_2_ (-18.9 ± 17.8%) in Group C. The anxiety level showed no change after intervention. The menstrual blood volume reduced slightly after intervention and the change of menstrual blood loss in Group B was significant (*P* = .038).

**Conclusion:**

The rESWT applications on the abdominal acupuncture points safely and effectively reduced the menstrual pain, which was not associated with the prostaglandin changes. The rESWT-reduced pain seemed equally effective with the intervention applied during the follicular phase or luteal phase of the menstrual cycle. Heat patch placed on the abdominal acupuncture points also reduced the pain severity and duration, indicating that the improved blood flow could effectively alleviate the menstrual pain with PD. The changes in anxiety level and menstrual blood loss were slight after intervention.

## 1 Introduction

Dysmenorrhea is one of the most common gynecological conditions in women.^[1]^ Primary dysmenorrhea (PD), defined as menstrual pain without any structural lesions, usually begins shortly before or immediately after the onset of the menstrual cycle and commonly lasts for 48 to 72 hour.^[2]^ It is accompanied by general symptoms, such as nausea and vomiting, malaise, weakness, lower backache, and diarrhea.^[2]^ In ovulatory cycles, women secrete high levels of or have increased sensitivity to prostaglandins.^[3]^ These prostaglandins may stimulate myometrial contractions and sensitize pain fibers, and thus induce pelvic pain. In general, non-steroidal anti-inflammatory drugs and oral contraceptives are the first choice of treatment for reducing moderate-to-severe pain in women with PD.^[4,5]^ However, these medicines have the side effects in the long run. Therefore, alternative therapies, such as transcutaneous electrical nerve stimulation and heat intervention, have been advocated as major non-pharmacological interventions for PD.^[6–8]^ In particular, young women in China with PD prefer to select traditional Chinese medicine for pain relief, namely acupuncture and moxibustion.^[9]^ However, this intervention needs special preparation and takes more time. A non-invasive, more effective method to relieve menstrual pain and cramps in women with PD remains to be verified.^[10,11]^

Radial extracorporeal shock wave therapy (rESWT) is a novel physical therapy in which a sequence of acoustic pulses characterized with high peak pressure (100 MPa), fast rate (<10 ns), short duration (10 μs), and low-energy density (from 0.003 to 0.890 mJ/mm^2^) is applied for treating a variety of chronic soft tissue pains.^[12]^ Numerous studies have demonstrated the efficacy of rESWT in the treatment of skeletomuscular pains associated with calcifying tendinitis, plantar fasciitis, and osteonecrosis of the femoral head.^[13,14]^ It is believed that rESWT on soft tissue enhances cell proliferation and inhibits substance P production.^[15]^ It is a safe, effective, and noninvasive method for alleviating pains, including low back pain.^[16]^ Recently, Li et al reported that the PD related pain could be reduced after one session of applying rESWT on three acupuncture points in the legs (Sanyinjiao, Guanyuan and Zusanli) in the luteal phase of the menstruation cycle in a group of young women.^[17]^ A putative mechanism proposed by the study was probably related to rESWT-altered secretion of prostaglandin and improved pain threshold as a result of electrical stimulation of the acupuncture points.^[17]^ However, questions remain whether application of rESWT intervention on the acupuncture points in the abdominal area and in different time during the menstrual cycle (ie, the follicular phase vs the luteal phase) would be safely and effectively affect the pain-release outcome.

The present study aimed to test the hypothesis that rESWT could be safely and effectively applied to reduce pain intensity and pain duration in young women with PD by stimulating the abdominal acupuncture points in both the follicular phase and the luteal phase of the menstrual cycle. This rESWT-induced effect was associated with reductions of prostaglandin F2alpha (PGF_2α_) and prostaglandin E2 (PGE_2_). The changes in anxiety level and menstrual blood loss were observed after three-cycle intervention.

## 2 Methods

### 2.1 Study design

This randomized trial (registered # NCT03121170) was approved by the Shanghai Xuhui Central Hospital Ethics Committee. The subjects’ enrollment started in February 2017 and the study intervention completed in June 2018. Fifty participants were recruited from Shanghai Xuhui Central Hospital and the nearby universities in the area after having signed an informed consent for participation in the trial.

### 2.2 Participants

All enrolled subjects provided a gynecological report prepared by the gynecologist who practiced the medicine in the field. The inclusion criteria included:

(1) clinically diagnosed with PD according to the Primary Dysmenorrhea Consensus Guideline^[18]^;
(2) age of 18 to 30 years^[19]^;
(3) regular menstrual cycle of 21 to 35 days in the recent three menses;
(4) having a pain intensity of 4 to 10 cm on a visual analog scale (VAS);
(5) willingness to participate in the clinical trial.

The exclusion criteria included:

(1) having any known chronic disease (such as cardiovascular or renal diseases) or secondary dysmenorrhea;
(2) being pregnant or planning to get pregnant any time during the trial;
(3) having received other treatments for PD in the past half year.

All enrolled participants were requested to maintain their daily routines/activities, lifestyles, and medications throughout the study.

**Figure 1:**
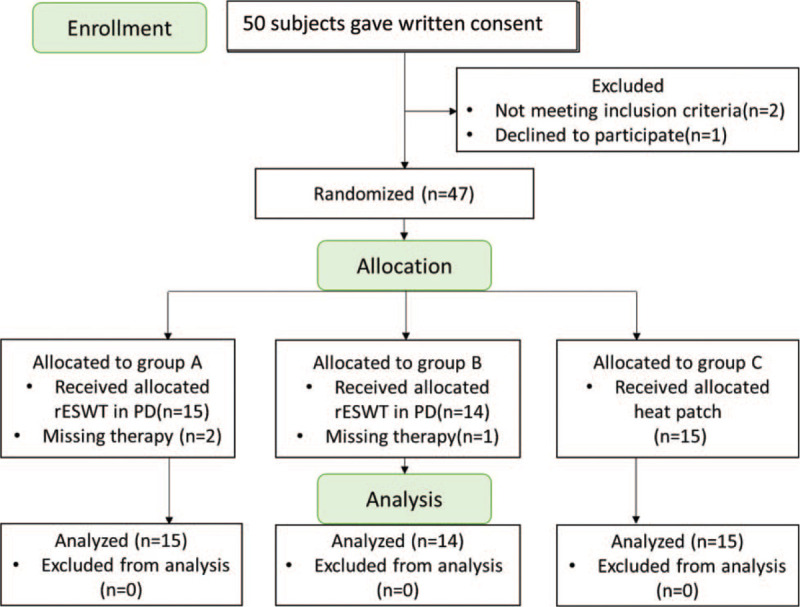
Enrollment of study participants.

Participants were then randomly assigned to one of the three groups, using a computer-generated random-allocation sequence by a research assistant who was independent to the trial team. Groups A and B were the treatment groups, and Group C served as the control (Fig. 1). The Group A participants received rESWT on the first (1–48 h) and third (48–96 hours) days of the menstrual cycle, that is, during the follicular phase.^[20]^ The Group B participants received rESWT on the third and fifth day of the last week of the menstrual cycle (the luteal phase), which was similar to the time period reported by Li Han et al.^[17]^ The Group C participants received the heat patches on the first 3 days of the menstrual cycle. The treatments (Groups A and B) with the rESWT application or with the heat patch intervention (Group C) lasted for three consecutive menstrual periods.

### 2.3 Interventions

A new rESWT device (Master puls MP100, Storz Medical, Tagerwilen, Switzerland) was used to treat the participants in Groups A and B. It uses the D-ACTOR technology to perform biomechanical stimulation of tense, shortened, or overstretched muscles and tendons with low-to-medium energy. The pressure algometer (Dolometer) has been used for the scientific examination of myofascial trigger points.^[21]^ Using this shock wave therapy device, radial shock wave is created ballistically with the pressurized air source accelerating a bullet to strike a metal applicator. The kinetic energy produced is transformed into radially expanding shock waves from the application site into the tissue to be treated.

**Figure 2:**
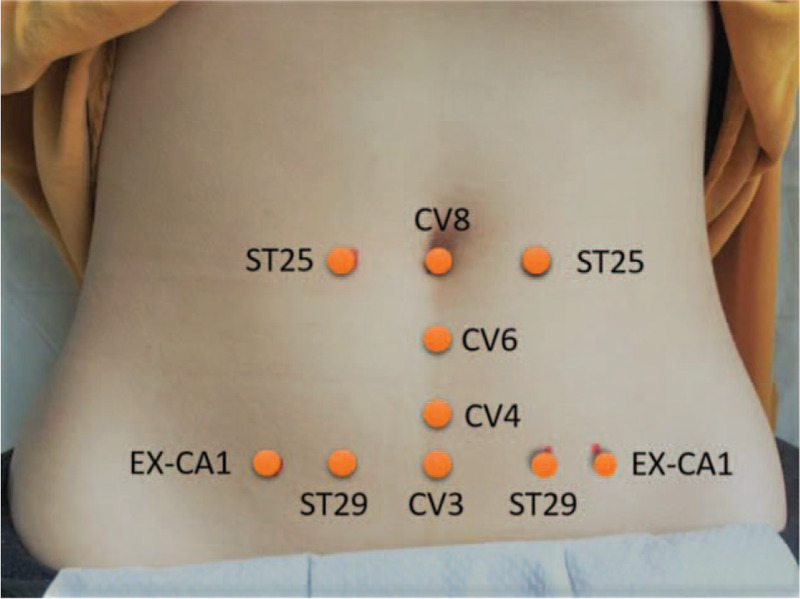
Acupuncture points on the lower abdomen. Ten acupuncture points selected for the treatment using radial extracorporeal shock wave therapy and heat patch.

The treatment was applied at the following 10 acupuncture points in sequence in the lower abdomen based on local points (Fig. 2): Shenque (CV8), Qihai (CV6), Guanyuan (CV4) and Zhongji (CV3) at the center, and Tianshu (ST25), Guilai (ST29), and Zigong (EX–CA1) at the right and left sides of the lower abdomen.^[22]^ Each acupuncture point was shocked 300–400 times using the rESWT device at the frequency of 15 hertz, pressure of 1.8 to 2.2 bar, and the total number of pulses to each acupuncture point were approximately 6000 which elicited a sensation called “De Qi” from participants’ subjective feeling.^[23]^ The acupuncture points were covered with sufficient coupling agent so that the detector could move smoothly. It took approximately 10 min for each treatment session. All participant received two treatment sessions within one menstrual cycle and totally six treatments for the intervention. All treatments were conducted by a medical doctor or physical therapist who was trained before the trial.

The control group received heat therapy using heat patches (∼40^o^C, 7 × 12 cm) which have been proved convenient to elicit analgesic effect on PD without any side effects.^[6]^ Heat patches (Yancheng Aize Biological Technology Co., Ltd. Shanghai, China) were applied on the acupuncture points simultaneously (see Fig. 2) for approximately 8-10 hours per session by the participants during the first 3 days of their menstrual cycle. All participants were trained to place heat patches by themselves on the acupuncture points. They received a remind phone call for every scheduled intervention.

### 2.4 Outcome measurements

The primary outcomes of measurements were the pain severity scores from VAS and the duration of pain (in hours). A 10-cm (from 0 to 10) VAS was used to score pain severity, where 0 indicated no pain and 10 was the most severe pain, and was assessed by the participants during the menstruation before the intervention (ie, baseline). In addition, the duration of pain was recorded at the same time. At the end of every menstrual cycle following the interventions, both VAS for pain severity and the duration of pain of all participants were asked over telephone or face-to-face and recorded by the investigators.

The secondary outcomes of measurements were blood plasma prostaglandin F2alpha (PGF_2α_; YM Company, Shanghai, KJ0161) and prostaglandin E2 (PGE_2_; R&D Systems Inc., Shanghai, KGE004B) levels. Once menstrual bleeding started, blood extraction was performed by the experienced nurses 24 to 48 hour before the interventions as the baseline and 24 to 48 hour after the last session of the intervention. Blood samples were centrifuged for 10 min to separate plasma and were frozen at −80 °C until the batch analyses after the completion of the study interventions. The plasma samples were masked with the interventions and analyzed using enzyme-linked immunosorbent assay (ELISA) according to the manufacturer's instruction by the technicians not involved in the research project.

Other outcomes of measurement were self-rating anxiety scale (SAS) and pictorial blood loss assessment chart (PBAC). The charts would be completed by participants before intervention and after intervention. SAS was measured since females have emotional changes before and during menstruation. PBAC is a semiquantitative and simple method to assess menstrual blood loss. Participants can directly record the number of their used sanitary pads and degree to which they are bloodstained.^[24]^ The final scores would be calculated by the investigators.

### 2.5 Sample size calculation

The sample size was calculated using an equation of repeated measures design (two-factor). Accordingly, a sample size of 42 participants was considered to be adequate to detect an effect size of 0.25, with a power of 80%, number of groups of 3, number of measurements of 2 using G∗power software (version 3.1). We expected the participant dropout rate to be as high as 20% and so decided to recruit a total of 50 participants.

### 2.6 Statistical analysis

Baseline characteristics of the participants among the different groups were compared using one-factor analysis of variance (ANOVA) for continuous variables and Chi-square test for categorical variables. Two-factor of ANOVA was applied to test the influences of group-factor and time-factor (before vs after) on the primary and secondary outcomes. If a major factor showed significance (*P* *<* .05), the post-hoc Tukey's analysis was conducted. Paired *t*-test or Wilcoxon signed-rank test (if the data failed a normality test) was conducted to determine a significance within-group changes before (ie, baseline) vs after intervention. In addition, the intervention-induced relative change (%) from the baseline was analyzed using one-factor ANOVA to determine the difference in the changes among the groups. Data present as group means ± standard deviation (SD) of the means. Statistical analyses were performed using the statistical package of SPSS (version 24.0, IBM).

## 3 Results

During the study period, 50 participants with PD were admitted and gave wrote consent forms. Three of them were excluded because of exclusion criteria and decline to participate. Three participants missed the therapy during intervention, so a final population sample of 44 participants was completed the study.

Table 1 summarizes the participants’ age, physical and medical conditions, and occupations. None of these characteristics was statistically different among the groups.

**Table 1:** Characteristics of the study participants.

Characteristic	Group A (n = 15)	Group B (n = 14)	Group C (n = 15)	*P* value
Age (yr)	23.9 ± 2.5	23.5 ± 2.8	22.8 ± 1.8	.831
Age of PD onset (yr)	17.1 ± 3.1	16.1 ± 2.9	15.5 ± 2.4	.193
Height (cm)	161 ± 5	163 ± 5	165 ± 5	.142
Weight (kg)	50.13 (5.62)	52.28 (7.81)	56.33 (6.59)	.148
BMI (kg/m^2^)	19.25 (2.27)	19.65 (1.96)	20.60 (1.76)	.494
Treatment, percentage				.488
NSAIDs (other than aspirin)	33.33%	35.71%	40.00%	
Acupressure	20.00%	21.43%	26.67%	
Other treatments	46.67%	42.86%	33.33%	
Occupation, percentage				.096
Students	60.00%	50.00%	66.67%	
White-collar workers	40.00%	42.86%	26.67%	
Others	0	7.14%	6.67%	

**Figure 3:**
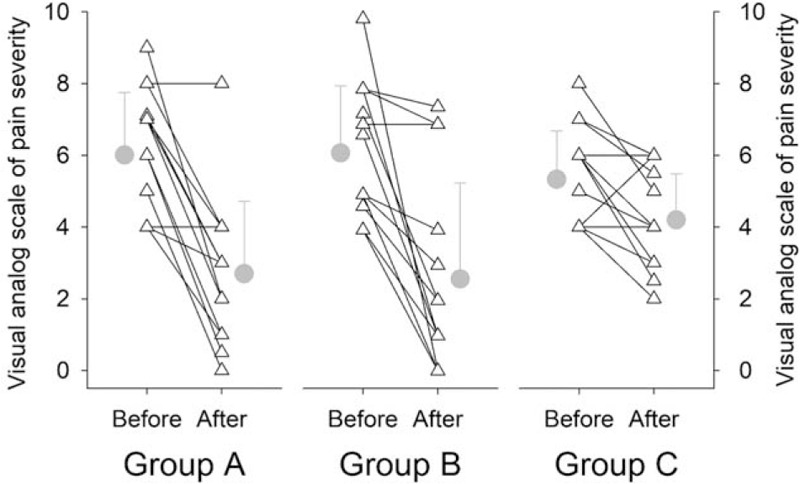
Pain severity before and after interventions. The pain severity scores from visual analog scale are not different among the groups (group-factor *P* = .669), but is significant different before vs after interventions (time-factor *P* < .001). Although all interventions significantly reduce the pain severity scores, this intervention-induced change is more significant (*P* = .003) in Group A and Group B as compared with Group C. There is no difference in the intervention-induced changes between Group A and Group B. Triangle symbol indicates the individual data; gray circle symbol represents group mean with standard deviation.

**Figure 4:**
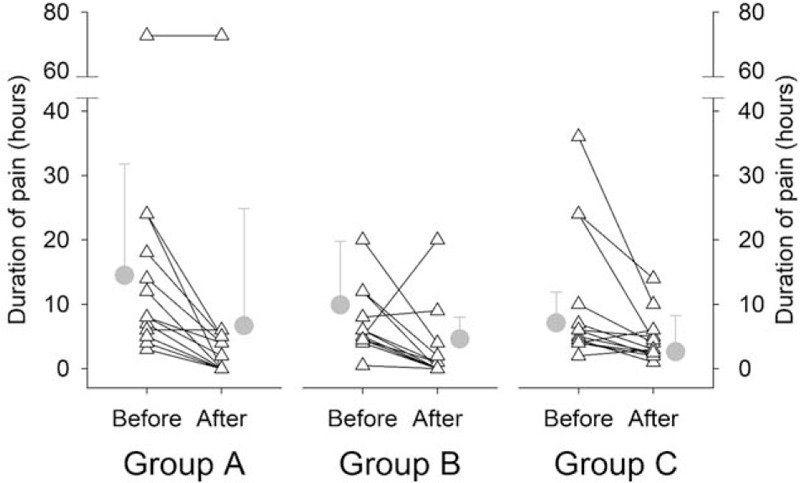
Pain duration before and after interventions. The pain durations are not different among the 3 groups (group-factor *P* = .169), which are significantly affected by the interventions (time-factor *P* = .018). The group means of the pain durations are 14.5 ± 17.3, 7.1 ± 4.8 and 9.9 ± 9.9 hours in Groups A, B and C, respectively. The corresponding pain durations reduce to 6.7 ± 18.2, 2.6 ± 5.6 and 4.6 ± 3.4 hours in Groups A, B, and C, respectively, after the interventions. Triangle symbol indicates the individual data; gray circle symbol represents group mean with standard deviation.

Figure 3 illustrates the VAS scores for pain severity before and after interventions. The baseline scores were 6.0 ± 1.7, 6.2 ± 1.9 and 5.3 ± 1.3 in Groups A, B and C, respectively. Two-factor ANOVA suggested that group-factor was not significant (*P* = .669). However, the VAS scores after the interventions, i.e. 2.7 ± 2.0, 2.6 ± 2.7 and 4.2 ± 1.3 in Groups A, B and C, respectively, were significantly lower than the baseline in all three groups (time-factor *P* < .001). Post-hoc analysis indicated that the intervention-induced relative changes by the rESWT interventions in Groups A (-53.8 ± 33.7%) and B (-59.3 ± 36.7%) were more significant compared with that in Group C (-18.7 ± 27.1%); there was no difference between the two treatment groups. The duration of pain was not statistically affected by group-factor (*P* = .169); but significantly determined by time-factor (*P* = .018), see Figure 4. However, the intervention-induced relative changes were not statistically different among the groups, i.e., -74.9 ± 34.4%, -58.0 ± 107.2% and -36.3 ± 40.9% in Groups A, B and C, respectively.

Baseline PGF_2α_ concentration was significantly higher in Group B than Group A or Group C, which was significantly reduced only in Group B after the intervention (Table 2). PGE_2_ concentrations were not significantly different among the groups (group-factor *P* = .081), nor were significantly different before and after interventions (time-factor *P* = .553). The relative change in PGE_2_ was significant only in Group C (Table 2).

**Table 2:** PGF_2α_ and PGE_2_ levels between groups before and after the intervention.

	Time	Group A (n = 15)	Group B (n = 14)	Group C (n = 15)	*P* value
PGF_2α_ (pg/mL)	Baseline	459 ± 118	649 ± 248#	479 ± 123^∗^	.008 (group-factor)
	After	423 ± 94	468 ± 136^†^	455 ± 95	.014 (time-factor)
	% Change	−4 ± 26	-21 ± 33^†^	1 ± 33	.169
PGE_2_ (pg/mL)	Baseline	392 ± 104	498 ± 471	400 ± 70	.081 (group-factor)
	After	351 ± 7	520 ± 492	318 ± 58	.553 (time-factor)
	% Change	−7 ± 24	7 ± 29	−19 ± 18^†,∗^	.020

Table 3 presents the anxiety level and menstrual blood loss between groups before and after the intervention. The SAS data presented no significance before and after intervention. Menstrual blood loss in Group B decreased significantly (*P* = .038), while the menstrual blood loss in Groups A and C decreased slightly.

**Table 3:** SAS and PBAC between groups before and after the intervention.

	Time	Group A (n = 15)	Group B (n = 14)	Group C (n = 15)	*P* value
SAS	Baseline	35 ± 7	34 ± 3	34 ± 4	.777
	After	39 ± 10	41 ± 7	42 ± 8	.731
PBAC^∗^	Baseline	105 ± 70	141 ± 98	106 ± 72	–
	After	80 ± 58	103 ± 79^†^	93 ± 53	–

## 4 Discussion

The present study demonstrated that applications of radial extracorporeal shock wave therapy (rESWT) and heat patch on the acupuncture points in the lower abdomen significantly reduced the pain severity and pain duration during the menstrual cycle in young women with primary dysmenorrhea (PD). Intervention-reduced pain severity and pain duration were equally effective with the rESWT applications in the follicular phase or the luteal phase of the menstrual cycle. The mechanistic mediators for alleviating the PD related pain appeared not associated with the intervention-induced changes in PGF_2α_ or PGE_2_. The majority of participants decreased slightly their menstrual blood volume after intervention and had no emotional changes (anxiety level). There was no adverse event or unexpected incidence observed or reported during the trial.

It is proposed that the mechanism of causing menstrual pain is associated with the unbalanced or augmented levels of prostaglandins during menstruation ^[4,11]^. Menstrual cramps associated with augmented prostaglandin levels could cause visceral pain ^[25]^ and increase uterine pressure to elicit muscle spasm.^[26]^ Our data suggested that the PGE_2_ level was significantly decreased only in the Group C participants (the control group) with the heat therapy intervention (Table 2). However, the PGE_2_ levels in Groups A and B were not significantly altered after the rESWT treatments. Although the PGF_2α_ level in Group B was significantly reduced following the rESWT intervention, this difference in the PGF_2α_ response was probably related to a higher baseline level in Group B (Table 2). Therefore, this trial seemed to suggest that the intervention-reduced pain was not explained by the prostaglandin decreases and that a greater reduction of the pain severity with the rESWT treatments in Groups A and B than with the heat patch application in Group C was not determined by the different changes in PGF_2α_ and/or PGE_2_ in the present study.

The rESWT intervention produced shock waves could alleviate the regional muscle spasm by the fast delivery of low energy density and high frequency pressure through the acupuncture points in the lower abdomen. These pulsatile waves may stimulate the muscle nodules by activating myofascial release, modifying peripheral sensory nerve fibers and their conduction, and thereby decreasing dysmenorrhea to the uterus and alleviating menstrual pain.^[27,28]^ Furthermore, the process of the rESWT intervention could increase uterine blood flow ^[29]^ and then release the inflammation and irritation.^[30]^ This may be a plausible mechanism ^[17]^ for the outcome that the thermal intervention with heat patches on the abdomen could also effectively reduce the pain (Figs. 3 and 4). Furthermore, improved local circulation by the rESWT intervention or the heat stimulation seemed to explain a statistically similar and effective reduction of the pain duration in all three groups. This improved circulation could alleviate the blockade of blood flow during menstrual cycle.^[31,32]^ As compared to the Group C post-heat intervention, a greater reduction of the pain severity after the rESWT interventions in either Group A or Group B was more likely resulted from the rESWT- produced energy which was transmitted to the region, including the uterus, in addition to the increased local blood flow.

The present trial selected the acupuncture points in the abdomen which could lead to lower physical and emotional burdens during treatment.^[33,34]^ Previous studies have proven that the rESWT application on the acupuncture points can relieve pain and adjust energy balance with excellent long-term outcome.^[35]^ A recent report indicated that CV8 regulated neuroendocrine-immune network through mediating PGF_2α_.^[36]^ This rESWT application produces low-to-medium energy with a penetrative depth of up to 3 to5 cm (similar to the acupuncture needle penetration), and effectively stimulates the acupuncture points and reduces soreness.^[13]^ Since the probe of the rESWT device has a greater surface area, the energy could easily reach wider area and/or more muscles compared to the application of acupuncture needle. Additionally, Zhao et al illustrated that the level of anxiety and menstrual blood volume were not risk factors in PD based on sociodemographic information, lifestyle behavior and emotional characteristics, menstrual pattern information in China,^[37]^ which were consistent with our results. This may explain why the data of SAS and PBAC were showed slight changes in the study. Yet, there is seldom clear evidence to demonstrate the relationship between SAS or PBAC and menstrual pain through the shock wave treatment. So the future study may increase the shock wave times and use alkaline hematin (gold standard) to assess the menstrual blood loss to observe the impact of rESWT. Moreover, the similar reductions of the pain severity and pain duration observed in Group A and Group B suggested that the rESWT applications during the follicular phase or during the luteal phase were safe and equally effective (see Figs. 3 and 4). Therefore, the beneficial influences appeared not affected by the waxing and waning of the hormones during the different phases of the menstrual cycle.

A main limitation of the study is no measurements of the blood flow and the energy transmitted through the tissues/organs in the area during the interventions. Although PBAC was measured, and it does not assess the blood flow volume velocity. In addition, there is no follow-up assessment for the participants in the present trial. Therefore, it remains to be determined how long the intervention-reduced pain severity and duration can last after the completion of the interventions.

## 5 Conclusion

The present study suggests that the rESWT applications on the acupuncture points in the lower abdomen can safely and effectively reduce the menstrual pain, which is not explained by the changes in PGF_2α_ and PGE_2_. The rESWT-reduced pain seems equally effective when the intervention is applied during the follicular phase or the luteal phase of the menstrual cycle. Thermal stimulation by the heat patch placed on the abdominal acupuncture points also reduces the pain severity and pain duration, indicating that the improved blood flow in the abdominal area can effectively alleviate the menstrual pain in young women with primary dysmenorrhea. The changes in anxiety level and menstrual blood loss were slight after three-cycle intervention.

## Acknowledgments

We sincerely thank the participants for their cheerful cooperation and Dr. Wendy Yajun Huang and Dr. Xiangrong Shi for their help in proofreading the manuscript. Study data are available from the corresponding author on request. The authors declare that they have no competing interests.

## Author contributions

**Data curation:** Ruirui Xing, Yan Wang.

**Funding acquisition:** Jian Yang.

**Investigation:** Ruirui Xing, Jian Yang, Renwei Wang.

**Methodology:** Ruirui Xing, Jian Yang, Renwei Wang.

**Project administration:** Jian Yang.

**Resources:** Jian Yang, Yan Wang.

**Supervision:** Ruirui Xing, Jian Yang, Yan Wang.

**Writing – original draft:** Ruirui Xing, Renwei Wang.

**Writing – review & editing:** Ruirui Xing, Renwei Wang.
